# The impact of gut microbiota on insulin resistance in polycystic ovary syndrome: mechanisms and therapeutic prospects

**DOI:** 10.3389/fcimb.2026.1840605

**Published:** 2026-08-19

**Authors:** Yifan Gao, Ciyan Liu, Xiaoxia He

**Affiliations:** Department of Gynecology, The Affiliated Traditional Chinese Medicine Hospital of Southwest Medical University, Luzhou, Sichuan, China

**Keywords:** gut microbiota, insulin resistance, mechanisms, polycystic ovary syndrome, treatment

## Abstract

Polycystic ovary syndrome (PCOS) is a prevalent endocrine and metabolic disorder, with insulin resistance (IR) serving as its core pathophysiological feature and a key driver of long-term metabolic complications. Recent studies have revealed that the gut microbiota, as a critical environmental factor, plays a significant role in the pathogenesis of PCOS. The gut microbiota profoundly engages in the pathophysiology of PCOS-IR through multiple pathways and networked interactions. This review provides a comprehensive narrative synthesis of the latest evidence on gut microbiota dysbiosis in PCOS-IR, with a particular focus on its molecular mechanisms, and evaluates therapeutic strategies based on gut microbiota modulation. It seeks to provide theoretical foundations and forward-looking perspectives for precision microbiome therapies. Importantly, this is a narrative review with a mechanistic focus, rather than a formal systematic review or meta-analysis.

## 1 Introduction

Polycystic ovary syndrome (PCOS) is one of the most common gynecological endocrine disorders and is widely recognized as a major cause of infertility (Sahmay et al., 2018). Globally, it affects 5% to 20% of women of reproductive age. The age-standardized prevalence rate, incidence rate, and disability-adjusted life years have all increased significantly worldwide, with notable variations across regions and countries (Azziz, 2018; Liu et al., 2025).

PCOS remains a syndrome, and thus no single diagnostic criterion (such as hyperandrogenism or polycystic ovaries) is sufficient for clinical diagnosis. The most widely adopted international standard is the 2003 Rotterdam criteria (Rotterdam ESHRE/ASRM-Sponsored PCOS Consensus Workshop Group, 2004). Women with PCOS most commonly present with infertility, irregular menstruation, obesity, hirsutism, and/or other outward manifestations of hyperandrogenism, such as acne or hair loss (Lujan et al., 2008). Furthermore, PCOS is associated with increased risks of metabolic disorders, including insulin resistance (IR), impaired glucose tolerance, type 2 diabetes mellitus (T2DM), dyslipidemia, and cardiovascular disease (Rodbard and Jellinger, 2010; Macut et al., 2017; Dubey et al., 2024). Consequently, there is growing concern regarding the overall impact of PCOS on women’s health.

**Figure 1:**
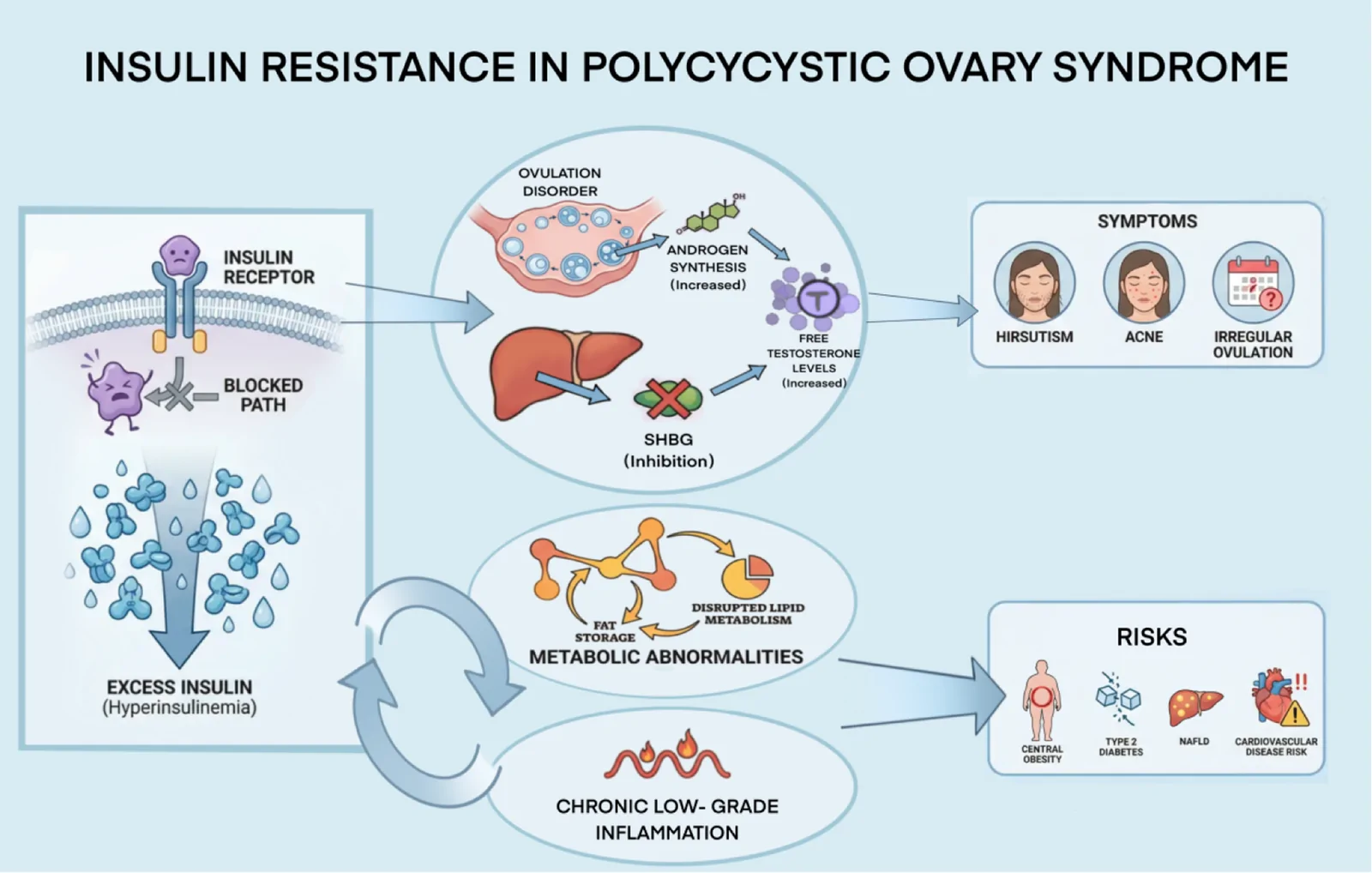
Insulin resistance in polycystic ovary syndrome. Elevated insulin promotes excessive ovarian androgen synthesis and inhibits hepatic sex hormone-binding globulin (SHBG) production, raising free testosterone and causing ovulatory dysfunction, hirsutism, and acne. Insulin resistance and hyperinsulinemia also trigger disrupted lipid metabolism, chronic low-grade inflammation, and metabolic abnormalities, increasing the risk of type 2 diabetes mellitus (T2DM), non-alcoholic fatty liver disease, and cardiovascular disease.

PCOS involves multiple pathophysiological factors, with 65% to 95% of women with PCOS exhibiting IR and compensatory hyperinsulinemia (HI) (Zhao et al., 2023). IR and the resulting hyperinsulinemia play a central role in the pathogenesis of polycystic ovary syndrome: on one hand, elevated insulin levels directly stimulate excessive androgen synthesis by ovarian theca cells (Sanchez-Garrido and Tena-Sempere, 2020; Zhang et al., 2020), and suppress hepatic production of sex hormone-binding globulin (SHBG), leading to elevated free testosterone (Samuel and Shulman, 2016; Deswal et al., 2018), This exacerbates hyperandrogenic symptoms such as hirsutism and acne, as well as ovulatory dysfunction. On the other hand, IR itself constitutes a fundamental metabolic abnormality. It synergistically contributes to lipid metabolism disorders, central obesity, and chronic low-grade inflammation, significantly increasing patients’ risk of developing type 2 diabetes, non-alcoholic fatty liver disease, and long-term cardiovascular disease (Diamanti-Kandarakis and Dunaif, 2012; O'Reilly et al., 2017; Zhao et al., 2023) (**Figure 1**).

**Figure 2:**
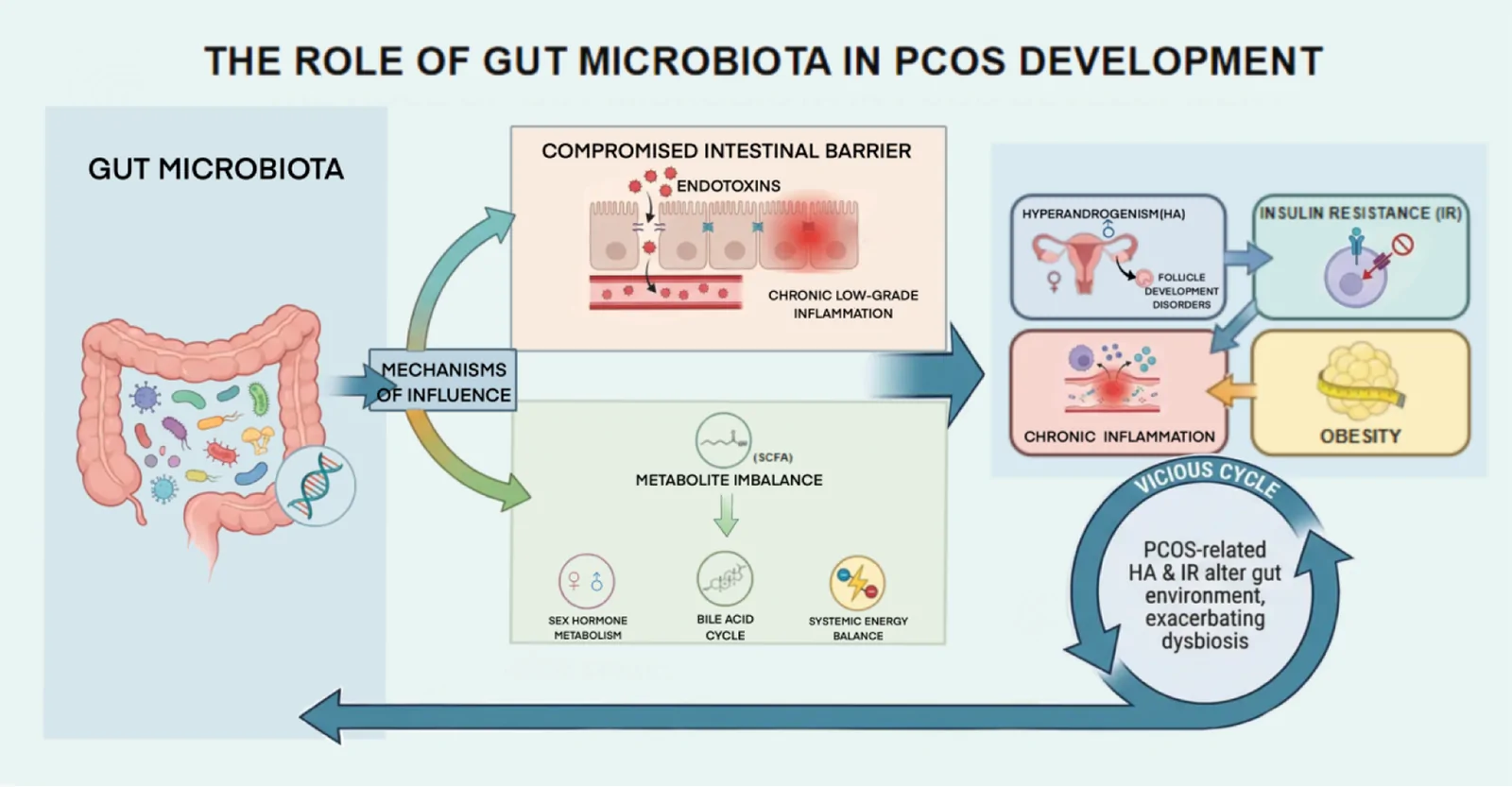
The role of gut microbiota in PCOS development. Gut dysbiosis compromises intestinal barrier function and induces endotoxin [e.g., lipopolysaccharide (LPS)] release, leading to hyperandrogenism (HA), insulin resistance (IR), chronic low-grade inflammation, follicular development disorders, and obesity. Imbalanced microbial metabolites disturb sex hormone metabolism, bile acid (BA) metabolism, and systemic energy balance. Hyperandrogenism and insulin resistance in turn alter the gut environment and exacerbate dysbiosis, forming a vicious cycle in the pathogenesis of polycystic ovary syndrome (PCOS).

The gut microbiota, often referred to as the human “second genome,” is closely associated with metabolic disorders, immune diseases, and infectious diseases (Liu et al., 2023). Recent studies have demonstrated that gut dysbiosis is linked to the onset and progression of PCOS, with the gut microbiota participating in the pathological pathways of PCOS, such as hyperandrogenism (HA), IR, chronic inflammation, and obesity (Chen and Pang, 2021; He and Li, 2021). Gut microbiota influence host metabolism and endocrine status through mechanisms like the “gut-ovarian axis”: dysbiotic microbiota can compromise intestinal barrier function, leading to endotoxin leakage into the bloodstream and chronic low-grade inflammation, thereby exacerbating IR (Liu et al., 2023). Concurrently, abnormalities in microbial metabolites disrupt sex hormone metabolism, bile acid cycling, and systemic energy balance, thereby promoting hyperandrogenism and impaired follicular development (Li et al., 2023; Salehi et al., 2024). Conversely, the hyperandrogenism and IR associated with PCOS also alter the gut environment, further exacerbating dysbiosis and creating a vicious cycle (Sun et al., 2023) (**Figure 2**).

Collectively, the available evidence suggests that gut microbiota dysbiosis may be more than a passive bystander in PCOS-IR. Rather, it could represent an important modifiable element in the disease network, and has therefore attracted attention as a potential target for future therapeutic strategies (Qi et al., 2019; Zhou et al., 2024). Based on this, this review provides a comprehensive narrative overview of the mechanisms by which gut microbiota influence PCOS-IR and the current applications of gut microbiota modulation strategies in improving clinical outcomes. The present work is designed as a mechanism-focused narrative review, not a formal systematic review, and aims to provide theoretical foundations and forward-looking perspectives for precision microbiome therapies.

## 2 Gut microbiota characteristics in PCOS patients

In recent years, multiple leading studies have demonstrated gut microbiota dysbiosis in both PCOS animal models and PCOS patients (Chen and Pang, 2021; Duan et al., 2021; He and Li, 2021; Yang et al., 2021). Reduced α diversity—the richness and evenness of species within a single sample—correlates with ecosystem vulnerability and functional decline, while β diversity reflects compositional differences between samples (Hills et al., 2019). The α diversity of gut microbiota in PCOS patients is typically significantly lower than in healthy controls, while β diversity reveals distinct overall microbial structures compared to non-PCOS populations. This disruption correlates with clinical manifestations of PCOS, such as IR, hyperandrogenism, and obesity severity (Liu et al., 2017; Torres et al., 2018; Zhang et al., 2019a; Rizk and Thackray, 2021; Zhu, 2024).

Beyond observational evidence of dysbiosis in PCOS patients, translational studies further confirm its pathogenic potential. Yang et al. successfully induced PCOS-like phenotypes, including IR, in germ-free mice by transplanting gut microbiota from PCOS women (Yang et al., 2021). Vrieze (Vrieze et al., 2012) observed increased body fat and impaired insulin response (IR) after transplanting healthy gut microbiota into germ-free mice. PCOS patients exhibit specific patterns of increase and decrease in gut microbiota at the phylum and genus levels (Liu et al., 2017; Torres et al., 2018; Qi et al., 2019; Zhou et al., 2020; Zhu et al., 2021) (**Table 1**).

**Table 1:** Alterations in gut microbiota composition in patients with polycystic ovary syndrome (PCOS).

Trend	Specific microbial group	Potential Functional Impact
Significant Increase (Harmful/Pro-inflammatory)	Relative abundance of Bacteroidetes often increases	Associated with high-fat diets and enhanced energy absorption; some species may promote inflammation.
	Prevotella	Certain species linked to mucosal inflammation and IR. Overgrowth observed in PCOS patients.
	Escherichia/Shigella	Opportunistic pathogens; their increase indicates potential inflammatory states and compromised barrier function.
Significant reduction (beneficial/anti-inflammatory)	The Firmicutes/Bacteroidetes ratio is often decreased	Changes in this ratio correlate with obesity and metabolic disorders. This ratio is frequently reduced in PCOS patients.
	Butyrate-producing bacteria: such as Propionibacterium and Ruminococcus	Produce butyrate and other SCFAs, serving as key beneficial bacteria for maintaining intestinal barrier integrity, anti-inflammation, and improving insulin sensitivity. Their reduction is a core feature of PCOS dysbiosis.
	Akkermansia	Considered the “next-generation probiotic,” it is closely associated with improved metabolic health and enhanced intestinal barrier function. Its abundance is typically reduced in PCOS patients.
	Bifidobacterium	A classic probiotic with anti-inflammatory and immunomodulating effects, its abundance is often diminished.

The information in this table is compiled from references (Liu et al., 2017; Torres et al., 2018; Qi et al., 2019; Zhou et al., 2020; Zhu et al., 2021).

In addition to the changes in taxonomic composition and α/β diversity, there is increasing evidence that the intestinal flora imbalance in PCOS patients is also reflected in multiple other dimensions. Metagenomic functional analysis showed that the gene pathways related to branched-chain amino acid biosynthesis, lipopolysaccharide (LPS) synthesis and oxidative stress response were significantly enriched in the microbiota of PCOS patients, while the gene pathways related to Short-Chain Fatty Acids (SCFAs) production (such as butyrate kinase, acetate CoA transferase) were generally missing (Liu et al., 2017; Zhu et al., 2021).

Beyond functional changes, the ecological architecture of the gut microbiota is also disrupted in PCOS. Co-occurrence network analysis showed that the network complexity of intestinal flora in PCOS patients decreased, the hub strains decreased, and the correlation between bacteria was looser, suggesting that the ecosystem vulnerability increased (Zhou et al., 2020). The time stability of the intestinal flora in PCOS patients is impaired, and the composition of the individual flora fluctuates greatly during the menstrual cycle, which may be driven by fluctuations in hormone levels (Rizk and Thackray, 2021).

Recent studies have noticed changes in non-bacterial components, including a decrease in the abundance of methanogens (such as Methanobrevibacter skrjabini) and intestinal fungal dysbiosis (such as an increase in Candida), which may be related to intestinal gas production and low-grade inflammation (He and Li, 2021). In summary, the virulence-related gene clusters (especially genes involved in LPS synthesis and bacterial motility) in PCOS patients were significantly enriched, which provided a genetic basis for the increase of endotoxemia potential (Yang et al., 2021). These extend our understanding of PCOS intestinal flora imbalance from simple composition changes to a broader functional and ecological level, suggesting that future research needs to adopt an integrated multi-omics approach.

## 3 Gut microbiota and the pathogenesis of PCOS-IR

**Figure 3:**
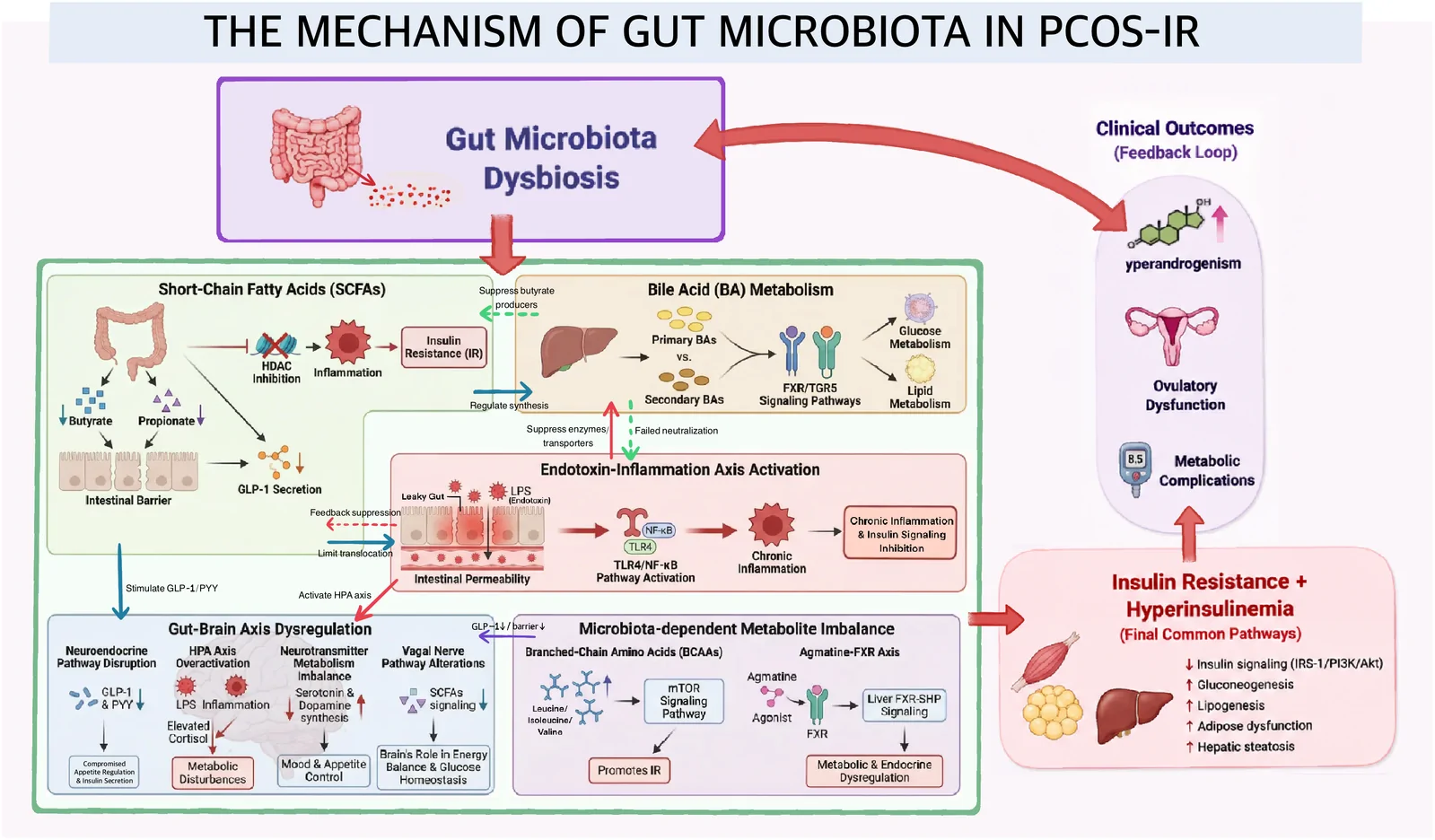
The mechanism of gut microbiota in PCOS-IR. Gut microbiota dysbiosis drives PCOS-IR through four interconnected pathways: (1) Reduced SCFAs impair intestinal barrier integrity via diminished HDAC inhibition, compromise GLP-1/PYY secretion, and promote inflammation. (2) Disrupted BA metabolism alters primary/secondary BA ratios, affecting FXR/TGR5 signaling and metabolic homeostasis. (3) Elevated BCAAs (leucine, isoleucine, valine) activate mTOR signaling, directly promoting IR. (4) Endotoxin-inflammation axis activation occurs when increased intestinal permeability allows LPS translocation, activating TLR4/NF-κB signaling and triggering chronic inflammation. These pathways converge on gut-brain axis dysregulation, including impaired GLP-1/PYY signaling, HPA axis overactivation, neurotransmitter imbalance, and altered vagal nerve signaling. The net result—IR and hyperinsulinemia—manifests through impaired IRS-1/PI3K/Akt signaling, dysregulated gluconeogenesis, lipogenesis, adipose dysfunction, and hepatic steatosis, culminating in hyperandrogenism, ovulatory dysfunction, and metabolic complications that form a vicious feedback loop perpetuating dysbiosis. BA, bile acid; BCAA, branched-chain amino acid; FXR, farnesoid X receptor; GLP-1, glucagon-like peptide-1; HDAC, histone deacetylase; HPA, hypothalamic-pituitary-adrenal; IR, insulin resistance; IRS-1, insulin receptor substrate-1; LPS, lipopolysaccharide; mTOR, mechanistic target of rapamycin; NF-κB, nuclear factor-kappa B; PI3K, phosphoinositide 3-kinase; PYY, peptide YY; SCFA, short-chain fatty acid; TLR4, Toll-like receptor 4; TGR5, G protein-coupled bile acid receptor 1.

The interconnected pathways through which gut microbiota dysbiosis contributes to PCOS-IR are summarized in **Figure 3**.

### 3.1 Short-chain fatty acids

Primarily comprising acetate, propionate, and butyrate, SCFAs are widely recognized as key messenger molecules mediating host-microbiota communication, playing a central role in maintaining metabolic homeostasis and immune balance. As an energy source for intestinal epithelial cells, SCFAs play a vital role in reducing inflammation and maintaining intestinal barrier function by stimulating the synthesis and release of phagocytic molecules, anti-inflammatory cytokines, chemokines, and protective peptides. In rodent models, SCFAs have been shown to stimulate the hypothalamic-pituitary-ovarian axis, enhancing intestinal immunity and alleviating PCOS-like phenotypes (He et al., 2022). In humans, correlational studies indicate that higher fecal SCFAs concentrations are associated with improved metabolic parameters, although direct causal evidence remains limited. PCOS patients exhibit altered gut microbiota diversity, leading to changes in SCFAs production that negatively impact host metabolism and intestinal barrier integrity. A meta-analysis (Jiao et al., 2018) demonstrated that high-fat diets induce increased acetate production in rodents, stimulating pancreatic cells to secrete more insulin via the parasympathetic nervous system. Concurrently, this promotes secretion of gastrin and ghrelin, leading to increased food intake. This feedback loop contributes to obesity and IR, participating in PCOS pathogenesis. Rekha K’s research indicates that SCFAs, particularly butyrate, act as potent histone deacetylase (HDAC) inhibitors. They suppress pro-inflammatory cytokine production (e.g., TNF-α, IL-6) through epigenetic regulation and modulate the differentiation and function of regulatory T cells (Tregs). The reduction of SCFAs in PCOS weakens this anti-inflammatory barrier, exacerbating the chronic low-grade inflammatory state closely associated with IR (Rekha et al., 2024). In summary, experimental evidence supports that SCFAs augmentation contributes to maintenance of intestinal barrier function and improvement of IR in animal models. Human interventional studies, though still limited, suggest that dietary strategies boosting SCFAs production may offer metabolic benefits in PCOS-IR patients, warranting further validation.

### 3.2 Bile acid metabolism

BA are metabolic products of cholesterol synthesized in the liver and subsequently degraded in the intestine. They function as signaling molecules regulating glucose metabolism and enhancing insulin sensitivity, primarily mediated by the farnesoid X receptor (FXR) and G protein-coupled BA receptor 1 to maintain metabolic homeostasis. BA serve as physiological ligands for FXR, binding to regulate various metabolic disorders. PCOS patients exhibit altered BA metabolism, affecting the conversion of primary to secondary BA. For example, PCOS patients exhibit elevated serum levels of primary BA (e.g., TCA and TDCA) and decreased levels of secondary BA (e.g., GDCA and GLCA) (Wang et al., 2026). Studies indicate that the gut microbiota influences the FXR-fibroblast growth factor 15 (FGF15) axis in mice (Degirolamo et al., 2014).

The most representative microorganisms in the human gut microbiota are bacteria, with the two most prevalent phyla being Bacteroidetes and Firmicutes. The gut microbiota exacerbates PCOS-related glucose and lipid metabolism disorders through the Bacteroides-BA-intestine-FXR signaling pathway. Bacteroides downregulate BA metabolic pathways, impairing the ability of intestinal lymphocytes to respond to secondary BA stimulation. This leads to insufficient secretion of anti-inflammatory factors, manifesting as varying degrees of clinical symptoms of PCOS-IR (Yang et al., 2021).

Chenodeoxycholic acid (CDCA), a BA with cholesterol-regulating, anti-inflammatory, and gut barrier-protective functions, shows potential therapeutic utility in PCOS management (Zhu et al., 2024). Experimental data from Qi (Qi et al., 2019) indicate that B. vulgatus negatively correlates with GDCA and TUDCA levels. B. vulgatus is significantly upregulated in the gut microbiota of PCOS women, affecting the metabolism of GDCA and TUDCA in the host. Chenodeoxycholic acid induces interleukin-22 (IL-22) secretion by gut group 3 innate lymphoid cells via GATA binding protein 3, and IL-22 subsequently improves PCOS phenotypes. Whether this pathway operates similarly in humans and contributes meaningfully to PCOS pathophysiology requires further investigation.

In summary, BA metabolism influences changes in glucose and lipid metabolism in PCOS patients. Certain BA exhibit insulin-reducing effects, offering novel therapeutic insights for the clinical management of PCOS-related IR. It is worth noting that, in addition to the BA pathway, certain microbial metabolites (such as agmatine) can also directly target FXR; see Section 3.4.2 for details.

### 3.3 Activation of the endotoxin-inflammation axis

Dysbiosis of the gut microbiota increases intestinal mucosal permeability, allowing endotoxins to enter the bloodstream through damaged intestinal epithelium. This affects multiple organs and systems, contributing to the development of “leaky gut syndrome” (Aleman et al., 2023). LPS is a major component of intestinal Gram-negative bacteria and a key initiator of the endotoxin-inflammation axis. In animal models and *in vitro* studies, LPS translocated across a compromised intestinal barrier activates Toll-like receptor 4 (TLR4)-mediated inflammatory signaling, which has been linked to impaired insulin receptor signaling.

Consequently, antibiotic suppression of Gram-negative rods to reduce LPS entry into the bloodstream has been demonstrated to improve insulin sensitivity in animal models (Tremellen and Pearce, 2012; Li et al., 2025). LPS absorbed into the bloodstream binds to LPS binding protein (LBP) and binds to TLR4 on innate immune cell surfaces. Following LPS-TLR4/myeloid differentiation factor 2 (MD-2) complex formation, signaling through myeloid differentiation primary response protein 88 and interleukin-1 receptor-associated kinase (IRAK), rapidly activating the nuclear factor kappa-B (NF-κB) and mitogen-activated protein kinase (MAPK, e.g., p38, JNK) pathways. This leads to the production of cytokines such as tumor necrosis factor-alpha (TNF-α) and interleukin-6 (IL-6) (Page et al., 2022).

Cani first proposed that endotoxemia generated by the gut microbiota may be a key factor triggering inflammatory activity leading to PCOS-IR (Cani et al., 2007). Studies show that serum LPS and LBP levels in PCOS women are higher than in healthy controls, and endotoxemia marker levels positively correlate with the IR index (HOMA-IR). This suggests endotoxemia markers are influenced by PCOS, with elevated LPS exacerbating PCOS-IR patients. Significant alterations in serum endotoxemia markers indicate a correlation between endotoxemia and PCOS-IR (Banaszewska et al., 2020).

Zhao (Zhao et al., 2022) observed markedly elevated serum LPS levels in a letrozole-induced PCOS rat model, accompanied by upregulation of TLR4 and NF-κB p65 protein expression in ovarian and adipose tissues, along with increased release of key proinflammatory cytokines (e.g., TNF-α, IL-6). Experimental studies indicate that targeted inhibition of LPS-triggered NF-κB/MAPK signaling pathways (e.g., using berberine) effectively improves metabolic and endocrine abnormalities in PCOS models. Collectively, the experimental and clinical evidence supports a model in which gut dysbiosis-associated LPS elevation—particularly in the context of increased intestinal permeability—may contribute to systemic inflammation and insulin resistance. However, the directionality of these relationships in human PCOS remains to be definitively established, and the relative contribution of LPS versus other microbial and host factors awaits further elucidation.

### 3.4 Dysregulation of microbiota-dependent metabolites: branched-chain amino acids and agmatine compounds

#### 3.4.1 Branched-chain amino acids

Branched-chain amino acids (BCAAs) are essential amino acids that cannot be synthesized by the human body. They play a significant role in metabolic alterations associated with polycystic ovary syndrome (PCOS), potentially contributing to its pathogenesis and affecting female metabolic and reproductive functions. Mechanistic studies in rodents and cell lines suggest that BCAA excess may disrupt glucose homeostasis via mTORC1 activation, mitochondrial stress, and pro-inflammatory gene expression (Jia and Li, 2024). Whether these mechanisms operate as primary drivers of PCOS-IR patients, or rather as secondary metabolic consequences, is not yet resolved. Zhang (Zhang et al., 2014) found significantly elevated levels of leucine, glutamic acid, and valine in follicular fluid from 26 PCOS-IR patients. Hajitarkhani (Hajitarkhani et al., 2021) compared antiandrogen samples from 13 PCOS patients and 6 non-PCOS women, revealing a decreased branched-chain amino acid to aromatic amino acid ratio accompanied by elevated valine and leucine levels, directly associated with PCOS pathogenesis. Paczkowska (Paczkowska et al., 2023) conducted a case-control study (209 confirmed PCOS patients vs. 117 healthy controls) and found significantly higher serum concentrations of BCAAs, phenylalanine, and tyrosine in PCOS women compared to healthy controls, while tryptophan levels showed no difference between groups. Elevated levels of BCAAs (particularly leucine and isoleucine) and tyrosine are associated with PCOS-IR.

#### 3.4.2 Discovery of the agmatine-FXR axis

It is worth noting that, as described in Section 3.2 above, the gut microbiota (including the Bacteroidetes phylum) can influence the FXR signaling pathway by regulating BA metabolism, typically resulting in downregulation of FXR activity mediated by changes in the BA pool. However, agmatine, a metabolite produced by specific Bacteroides species (such as B. vulgatus) as described in this section, directly activates FXR via an atypical mechanism independent of BA. The former represents the gut microbiota’s overall remodeling of the BA pool, which typically influences FXR signaling indirectly; the latter constitutes a direct, non-BA-mediated FXR activation mechanism. These two mechanisms reflect the gut microbiota’s ability to regulate FXR activity in opposite directions via distinct molecular pathways, which may depend on microbial composition, local metabolite concentrations, and tissue-specific environments, thereby illustrating the complexity and networked nature of microbiota-host interactions.

Research by Yun C et al. indicates that B. vulgatus induces PCOS-like symptoms through its metabolite agmatine (derived from arginine via arginine decarboxylase). Agmatine activates the FXR pathway, subsequently inhibiting L-cell secretion of glucagon-like peptide-1 (GLP-1), leading to IR and ovarian dysfunction. The GLP-1 receptor agonist liraglutide and the arginine decarboxylase inhibitor difluoromethylginin alleviate ovarian dysfunction in a PCOS-like mouse model (Yun et al., 2024). While these findings establish agmatine as a candidate pathogenic factor in mice, translation to human PCOS—particularly regarding the relative contribution of agmatine versus other FXR ligands (e.g., BA)—requires further investigation in patient cohorts.

### 3.5 Dysregulation of the gut-brain axis

The gut microbiota maintains continuous bidirectional communication with the central nervous system through the complex “gut-brain axis,” a pathway involving neural, endocrine, immune, and metabolic signaling (Lee et al., 2023; Zhuang and Zhang, 2024). In the context of PCOS, it is hypothesized that microbial alterations may contribute to IR and reproductive dysfunction through disrupted neuroendocrine signaling, partially explaining the common psychosocial comorbidities observed in PCOS patients (Cani and Knauf, 2016; Torres-Fuentes et al., 2017). however, human data supporting this specific pathway in PCOS remain preliminary, and most evidence derives from rodent models.

#### 3.5.1 Neuroendocrine pathway dysregulation: diminished GLP-1 and PYY signaling

Intestinal L cells secrete key enteric hormones—GLP-1 and peptide YY (PYY)—in response to microbial metabolites such as SCFAs (Tolhurst et al., 2012; Psichas et al., 2015). Under normal conditions, GLP-1 and PYY synergistically act through the “gut-brain-peripheral organ” axis to: promote insulin secretion (the “incretin effect” of GLP-1); inhibit gastric emptying and glucagon secretion, thereby precisely regulating postprandial blood glucose (Baggio and Drucker, 2007; Holst, 2007). PCOS patients commonly exhibit reduced SCFAs production, potentially leading to decreased baseline or peak postprandial secretion of GLP-1 and PYY. Clinical studies indicate that some women with PCOS exhibit blunted GLP-1 responsiveness (Pontikis et al., 2011; Lin et al., 2015). This directly impairs their capacity to stimulate insulin secretion and suppress appetite, exacerbating hyperglycemia and obesity risks, thereby creating a vicious cycle.

#### 3.5.2 Hyperactivation of the hypothalamic-pituitary-adrenal axis

Persistent metabolic endotoxemia (elevated LPS) and chronic low-grade inflammation (e.g., IL-6, TNF-α) in PCOS are perceived by the brain as a chronic stressor. This leads to sustained HPA axis activation, prompting the adrenal cortex to secrete excessive cortisol (Silverman and Sternberg, 2012). Chronic elevated cortisol levels may promote hepatic gluconeogenesis, increasing blood glucose; antagonize insulin action, exacerbating peripheral IR; and drive visceral fat accumulation, altering body fat distribution (Kuo et al., 2015).

Concurrently, elevated cortisol indirectly disrupts the luteinizing hormone (LH)/follicle-stimulating hormone (FSH) balance by suppressing pulsatile secretion of gonadotropin-releasing hormone (GnRH) in the hypothalamus, thereby exacerbating ovulatory dysfunction (Whirledge and Cidlowski, 2010).

#### 3.5.3 Neurotransmitter metabolic dysregulation and psychiatric comorbidity

The gut microbiota is termed the “second brain” due to its capacity to synthesize or regulate precursors of multiple neurotransmitters (Góralczyk-Bińkowska et al., 2022). Over 90% of serotonin is synthesized in the gut by enterochromaffin cells, and the metabolic pathway of its precursor, tryptophan, is tightly regulated by the gut microbiota (Gershon and Tack, 2007). Dysbiosis may shift tryptophan metabolism toward the kynurenine pathway, producing neurotoxic byproducts like quinolinic acid while reducing serotonin synthesis (Agus et al., 2018; Roth et al., 2021). Serotonin deficiency is closely associated with depression, anxiety, and abnormal appetite regulation (Correia and Vale, 2022). Notably, PCOS patients exhibit significantly higher prevalence of depression and anxiety compared to the general population (Cooney et al., 2017). Zhu (Zhu et al., 2021) demonstrated altered abundance of gut microbiota genes associated with tryptophan metabolism.

Certain gut bacteria (e.g., specific Lactobacillus and Bifidobacterium species) can directly produce dopamine precursors or GABA itself. These neurotransmitters profoundly influence motivation, reward, mood, and stress responses (Asano et al., 2012; Cryan et al., 2019). Their dysregulation may be linked to the significantly higher prevalence of anhedonia and mood swings in PCOS patients (Cooney et al., 2017), as well as their tendency toward addiction to high-sugar, high-fat foods (Lee et al., 2017; Cooney et al., 2024). This abnormal eating behavior further exacerbates IR and metabolic status by increasing energy intake.

#### 3.5.4 Alterations in vagus nerve pathways

The vagus nerve detects mechanical and chemical stimuli within the gut, subsequently transmitting signals to the brain via afferent and efferent nerves, thereby influencing both bottom-up and top-down signaling pathways (Bonaz et al., 2018; Strandwitz et al., 2019). Metabolites such as SCFAs can activate receptors on vagal nerve endings in the gut (e.g., GPR41/43) (Karaki et al., 2008), transmitting signals to the solitary tract nucleus in the brainstem, which in turn influences the hypothalamic appetite center and autonomic nervous system output (Frost et al., 2014). The observed reduction of SCFAs in PCOS patients raises the possibility that this pathway is compromised, potentially contributing to impaired central regulation of energy balance and glucose homeostasis. However, this remains a hypothesis derived from animal studies and requires direct testing in human PCOS populations.

### 3.6 Interaction networks among SCFAs, BA, and LPS

The preceding sections have described the independent mechanisms of SCFAs, BA, and LPS in PCOS-IR; however, under pathophysiological conditions, these three factors do not act in isolation but rather form a complex interactive network through the “gut barrier-inflammation-metabolic signaling” axis, jointly driving the progression of insulin resistance.

SCFAs (particularly butyrate) serve as the primary energy source for colonic epithelial cells and can upregulate the expression of tight junction proteins (such as occludin, claudin-1, and ZO-1), thereby strengthening the intestinal mucosal barrier (Rekha et al., 2024). An intact barrier limits the entry of LPS from the intestinal lumen into the portal venous circulation via paracellular pathways. In PCOS, a reduction in SCFA-producing microbiota (such as Propionibacterium, Ruminococcus, and Akkermansia) compromises this barrier, promoting “leaky gut” and subsequent metabolic endotoxemia (Cani et al., 2007; Tremellen and Pearce, 2012). Conversely, restoring SCFAs levels through dietary fiber or probiotic supplementation reduces serum LPS concentrations and improves IR (Xue et al., 2019). Thus, SCFAs act as upstream negative regulators of the LPS-TLR4 inflammatory axis.

Recent evidence suggests that chronic low-grade endotoxemia can directly interfere with BA metabolism. LPS and pro-inflammatory cytokines (such as TNF-α and IL-6) can suppress the expression of hepatic cytochrome P450 enzymes (such as CYP7A1 and CYP27A1), which are rate-limiting enzymes in both the classical and alternative BA synthesis pathways (Degirolamo et al., 2014). This suppression alters the composition of the BA pool, reducing secondary BA and elevating certain primary BA—a feature that has been reported in patients with PCOS (Wang et al., 2026). Furthermore, LPS-induced inflammation can attenuate FXR-fibroblast growth factor 19 signaling, reducing negative feedback inhibition of BA synthesis in the liver and further exacerbating dysregulation of glucose and lipid metabolism (Qi et al., 2019). Thus, LPS-driven inflammation can secondarily disrupt BA homeostasis, aggravating the metabolic phenotype of PCOS-IR.

BA are not only substrates for microbial metabolism but also possess potent antibacterial effects themselves. The dysregulated BA profile in PCOS (such as elevated primary BA and reduced secondary BA) can reshape the composition of the gut microbiota. For example, CDCA can inhibit the overgrowth of Gram-negative opportunistic pathogens (such as Escherichia coli and Shigella) while promoting the growth of butyrate-producing bacteria (MahmoudianDehkordi et al., 2022; Zhu et al., 2024). Conversely, supplementation with secondary BA (such as deoxycholic acid and lithocholic acid) increases the abundance of Bacteroides and Bifidobacterium, thereby enhancing SCFAs production and strengthening intestinal barrier function. This forms a bidirectional positive feedback loop: a healthy BA profile promotes SCFA-producing microbiota, while SCFAs, in turn, promote normal BA metabolism through both FXR-dependent and FXR-independent pathways.

Taken together, these interactions indicate that gut dysbiosis in PCOS initiates a self-perpetuating vicious cycle: a decline in SCFA-producing bacteria is accompanied by an increase in Gram-negative pathogens (such as Bacteroides, Prevotella, and Enterobacter-Shigella), leading to reduced SCFAs levels and increased LPS production. Reduced SCFAs impair tight junction integrity, promoting LPS translocation (metabolic endotoxemia). LPS activates the TLR4/NF-κB pathway, releasing pro-inflammatory cytokines that further impair hepatic BA synthesis and FXR signaling. Alterations in the BA profile (decreased secondary BA and increased primary BA) weaken FXR-mediated GLP-1 secretion, promote insulin resistance, and fail to support beneficial SCFA-producing bacteria. The progressive worsening of insulin resistance (IR), hyperandrogenism, and obesity further alters the intestinal environment (e.g., intestinal pH, transit time), exacerbating the pre-existing dysbiosis. This suggests that therapeutic strategies targeting a single pathway alone yield limited and transient effects. A multi-pronged approach, such as dietary fiber (to promote SCFAs), specific probiotics (to competitively inhibit LPS-producing bacteria), and FXR agonists (to correct BA signaling), may be more effective in breaking this vicious cycle and achieving sustained improvement in PCOS-IR.

## 4 Gut microbiota-based treatment for PCOS-IR

Despite the availability of insulin sensitizers and hormonal therapies, no definitive disease-modifying or microbiota-targeted treatment exists for PCOS-IR. Clinically, insulin sensitizers and hormonal contraceptives are commonly used as first-line therapies to alleviate patients’ clinical symptoms (Sparić et al., 2024; Panchpuri et al., 2026). However, these treatments exhibit limitations including adverse effects, suboptimal efficacy, poor long-term adherence, and contraindications in half of cases (Stańczak et al., 2024). The central role of gut microbiota in PCOS-IR offers novel therapeutic perspectives and targets.

### 4.1 Dietary and lifestyle interventions

#### 4.1.1 Dietary

Diet represents the most potent external factor shaping gut microbiota. Optimizing dietary composition serves as the foundational and primary strategy for treating PCOS-IR. Dietary habits—including caloric intake, macronutrient composition, and food quality—play a pivotal role in weight management, insulin sensitivity, and inflammatory markers among women with PCOS (Gautam et al., 2025).

Dietary modifications are significantly associated with reduced IR and improved body composition in PCOS patients, particularly benefiting rapid BMI reduction (Shang et al., 2020). A diet low in refined carbohydrates and high in fiber helps regulate blood glucose levels and improve insulin sensitivity. Previous studies have reported the effects of various dietary patterns on PCOS management, including the Mediterranean diet (Scannell et al., 2025), the DASH diet (Shang et al., 2020), and the ketogenic diet (KD) (Turetta et al., 2025; Arsenaki et al., 2026), among others. A high-fiber diet, characterized by increased intake of whole grains, legumes, vegetables, and fruits, effectively promotes the production of SCFAs (particularly butyrate). This enhances intestinal barrier function, suppresses inflammation, and improves insulin sensitivity (Mirabelli et al., 2020). Low-carbohydrate diets not only help balance sugar and fat metabolism but also improve hormone levels. They reshape the gut microbiota structure, particularly altering the Firmicutes/Bacteroides ratio, and regulate secondary BA metabolism and SCFAs production (Porchia et al., 2020; Wang et al., 2023).

#### 4.1.2 Lifestyle interventions

Exercise can improve insulin responsiveness and resistance indicators. Regular physical activity also enhances metabolic function, aids weight loss, achieves glucose homeostasis, balances hormone levels, and improves overall quality of life. Among all exercise modalities, aerobic exercise (AE) demonstrates superior efficacy in PCOS patients, significantly reducing anthropometric indicators and hyperandrogenism while improving quality of life (QOL) in women with polycystic ovary syndrome (Cowan et al., 2023; Gautam et al., 2025). Exercise also enhances gut microbial diversity, promotes beneficial bacterial growth, increases SCFAs production, improves nutrient utilization, modulates neuroendocrine pathways, and enhances intestinal barrier integrity (Wegierska et al., 2022; Varghese et al., 2024).

### 4.2 Probiotics and prebiotics

Probiotic and prebiotic supplements are among the most widely investigated microbiota-targeted interventions for PCOS, with multiple studies reporting associations between their use and improvements in IR and metabolic-endocrine parameters. However, the clinical evidence base remains preliminary, and the magnitude of benefit is still debated (Heshmati et al., 2019; Martinez Guevara et al., 2024; Maddirevula et al., 2025). At present, probiotics and prebiotics cannot be recommended as standard therapies for PCOS-IR in clinical practice. They should be viewed as complementary or experimental interventions that hold promise but require further validation through large-scale, multicenter, double-blind, placebo-controlled RCTs with standardized outcome measures and longer follow-up periods.

#### 4.2.1 Probiotics

Probiotics are found in fermented foods and possess antioxidant, antibacterial, and anti-inflammatory properties. They can improve metabolic parameters, regulate the gut microbiota environment, and repair the immune system. Popular bacterial genera used as probiotics include Lactobacillus, Bifidobacterium, and Enterococcus. Research by Shoaei T (Shoaei et al., 2015) demonstrated that PCOS patients experienced significantly reduced serum insulin levels after 8 weeks of probiotic supplementation. Zhang J (Zhang et al., 2019a) showed that PCOS patients exhibited significantly elevated levels of succinate and SCFAs metabolism after 4 weeks of probiotic treatment, accompanied by increased coenzyme and vitamin metabolism.

Miao C (Miao et al., 2021) conducted a meta-analysis selecting seven studies involving 486 patients. Results indicated that probiotic/synbiotic supplementation significantly reduced HOMA-IR (mean difference = –0.37; 95% confidence interval –0.69, –0.05) and serum insulin (standardized mean difference = –0.66; 95% confidence interval –1.19, –0.12). However, the overall quality of evidence was graded as low to moderate due to substantial heterogeneity in study designs, probiotic strains, dosages, and treatment durations. Furthermore, funnel plot analyses suggested the presence of publication bias, and most trials did not adequately report allocation concealment or implement intention-to-treat (ITT) analyses, raising the risk of overestimating the true effect size.

Although the aforementioned studies suggest that probiotics may improve PCOS-IR-related indicators, the current evidence has limitations. Some trials had insufficient sample sizes and limited statistical power, making it difficult to detect the true effect size. Additionally, there were significant differences among trials in terms of probiotic strains, dosages, intervention durations, and outcome measures, resulting in poor comparability of results and unstable conclusions from meta-analyses. Some studies did not implement adequate blinding or allocation concealment, did not conduct intention-to-treat (ITT) analyses, and most trials were funded by probiotic manufacturers, posing potential risks of selective reporting and publication bias.

#### 4.2.2 Prebiotics

Prebiotics are fermentable substances that alter the composition and activity of the host’s gut microbiota. Prebiotics include polyunsaturated fatty acids, polyphenols, and carbohydrates such as Omega-3 polyunsaturated fatty acids (Omega-3 PUFAs), inulin, and fructooligosaccharides. Prebiotics can selectively induce the growth and fermentation of beneficial gut bacteria, as well as prevent pathogen colonization through interactions with them.

Lu L (Lu et al., 2022a) found that total dietary omega-3 polyunsaturated fatty acids and their subtypes (EPA and DHA) long-chain omega-3 polyunsaturated fatty acids were significantly negatively correlated with HOMA-IR, alleviating IR by regulating the silent information regulator 1 (SIRT1) pathway. Lu L (Lu et al., 2022b) found that dietary and serum long-chain n-3 polyunsaturated fatty acids were inversely correlated with serum insulin, total testosterone, and high-sensitivity C-reactive protein (hs-CRP), and positively correlated with serum FSH and SHBG. Zhang H (Zhang et al., 2023) reported that omega-3 PUFAs possess the capacity to regulate gut microbiota and alleviate ovarian dysfunction, thereby preventing IR by reducing inflammation and fat deposition in insulin-sensitive tissues. Xue J (Xue et al., 2019) observed that after 21 days of inulin intervention in PCOS mice, Lactobacillus and Bifidobacterium increased, ovarian damage decreased, and LPS production decreased, effectively improving PCOS-IR. A 12-week randomized controlled trial by Ziaei R et al. demonstrated that supplementation with both high-performance inulin (HPI) and oligofructose-enriched inulin (OEI) positively impacted weight loss, serum total testosterone, free androgen index (FAI), serum SHBG, serum triglycerides, and clinical manifestations of PCOS (including menstrual irregularities) (Ziaei et al., 2024).

It should be noted that research on prebiotics shares similar methodological limitations with that on probiotics, including small sample sizes, short study durations, and high heterogeneity in outcome measures; the strength of the clinical evidence also requires further validation through high-quality RCTs.

In summary, as intervention strategies targeting the gut microbiota, probiotics and prebiotics have demonstrated potential in improving PCOS-IR. Exploring the development and clinical application of probiotic and prebiotic products is of great significance for the prevention and treatment of PCOS-IR. Future efforts should focus on conducting large-scale, multicenter, double-blind, placebo-controlled confirmatory trials, as well as investigating the optimal dosage, treatment duration, and personalized combination strategies for specific strains and formulations.

### 4.3 Fecal microbiota transplantation

Fecal microbiota transplantation (FMT) involves transferring processed fecal fluid from healthy human donors into a patient’s gut to restore their intestinal microbiota for treating certain diseases (Tkach et al., 2022). FMT activates the adaptive immune response in the gut via the TLR pathway, thereby accelerating immunoglobulin production and protecting the intestinal mucosa, while also regulating patients’ blood glucose and insulin sensitivity. Patients receiving donor FMT exhibited increased peripheral insulin sensitivity (glucose disappearance rate, RD) compared to placebo-controlled groups. In terms of short-term insulin sensitivity improvement, FMT recipients demonstrated increased relative abundance of Clostridium bromatum and Enterococcus faecalis, thereby enhancing insulin sensitivity (Vrieze et al., 2012; Kootte et al., 2017; de Groot et al., 2020).

Concurrently, de Groot P et al. observed significant increases in secondary BA (cholic acid) in plasma and feces of patients receiving donor FMT, with marked elevations in isocholic acid and deoxycholic acid in metabolic syndrome-R feces (de Groot et al., 2020). Yu EW (Yu et al., 2020) demonstrated that after 6 weeks of FMT capsule administration, most participants exhibited persistent alterations in gut microbiome composition without serious adverse events. However, no statistically significant differences were observed between the FMT and placebo groups regarding IR, body weight, or most metabolic markers. More substantial FMT transplants may be required to achieve systemic changes. Additionally, in many studies, the microbial composition of recipients and donors varies due to differences in region, ethnicity, gender, and diet.

Although FMT shows theoretical potential for improving metabolic parameters, we need to examine the key technical bottlenecks facing FMT. There are individual variations in the microbial composition of healthy donors, while the gut microbiome of patients with PCOS-IR exhibits unique dysbiosis characteristics. Currently, there are no donor stratification criteria based on PCOS-specific phenotypes; blindly transplanting unscreened donor fecal microbiota may lead to colonization failure or functional mismatch. Additionally, the colonization of exogenous microorganisms in the recipient’s gut is antagonized by factors such as the host’s native microbiota, diet, and immune status, resulting in poor long-term stability. Most studies have observed only short-term changes in the microbiome, with low long-term colonization rates and a tendency for microbial structure to revert to baseline levels. Multiple studies have shown that glycated hemoglobin (HbA1c) levels decrease slightly 6 weeks after FMT; however, the beneficial effects of FMT on RD and HbA1c are not sustained long-term. Metabolic and microbiome changes are short-lived, disappearing within 12 to 18 weeks after FMT administration (Kootte et al., 2017; Zhang et al., 2019b; Zecheng et al., 2023).

Furthermore, there is currently a lack of biomarkers to predict treatment response to FMT (such as baseline Akkermansia abundance and intestinal barrier function markers), making it difficult to effectively identify patient subgroups likely to benefit. Future research should focus on developing precision FMT strategies, such as functional screening of donors and combined pretreatment regimens to improve engraftment rates, as well as establishing multi-omics-based response prediction models. The impact of FMT on long-term clinical outcomes requires further investigation (Zhang et al., 2019b).In the specific context of PCOS-IR, no robust, PCOS-specific FMT trial has yet been published. The evidence discussed above is largely derived from studies on metabolic syndrome or type 2 diabetes, and its translatability to the unique endocrine and metabolic milieu of PCOS remains unknown. Consequently, FMT remains a highly experimental approach for PCOS-IR. Its potential should not overshadow the need for rigorous, disease-specific safety and efficacy trials before any clinical application can be contemplated.

### 4.4 Mechanism-specific interventions

Biomaterials or natural compounds capable of specifically binding and neutralizing LPS within the gut, preventing its translocation into the circulation. For instance, certain alkaline phosphatase preparations or specific polysaccharides have been studied for reducing metabolic endotoxemia. Therapeutic approaches may include using non-absorbable drugs that form complexes with LPS in the gut and reduce translocation, such as sevelamer (Page et al., 2022). Berberine, an active component of traditional Chinese medicine, has demonstrated clinically validated efficacy in improving PCOS-IR. Research indicates it not only modulates gut microbiota composition but also directly inhibits LPS-induced TLR4/NF-κB and MAPK inflammatory signaling pathways, thereby alleviating IR and ovarian inflammation (Izadparast et al., 2022).

Given FXR’s central role in integrating BA and agmatine signaling, developing non-BA selective FXR agonists (e.g., obeticholic acid) or Takeda G-coupled protein receptor 5 (TGR5) agonists represents a highly promising yet challenging new direction. However, precisely balancing their effects across different tissues (liver, intestine, immune cells) is essential—for instance, intestinal FXR activation is beneficial while hepatic overactivation may be harmful (Qi et al., 2019; Chen et al., 2024).Based on the groundbreaking discovery by Yun C, agmatine, as a microbiota-derived FXR agonist, has emerged as a highly promising new therapeutic target (Yun et al., 2024).

### 4.5 Synergistic therapy of drugs and microbiota

In the management of PCOS-IR, the combined use of traditional drugs and microbiota modulators represents a highly promising paradigm shift—transitioning from single-target interventions to integrated therapies targeting multiple systems and mechanisms (Cani et al., 2021).

Metformin, as a first-line treatment for PCOS-IR, traditionally functions by activating hepatic AMP-activated protein kinase (AMPK) to inhibit hepatic gluconeogenesis (Pernicova and Korbonits, 2014). Recent studies reveal that a significant portion of its broad clinical benefits—including weight loss and improved insulin sensitivity—are achieved through reshaping the gut-metabolic axis (Wu et al., 2017).

Multiple animal and clinical studies confirm that metformin significantly alters gut microbiota composition. Characteristic effects include increased abundance of Akkermansia muciniphila and certain SCFAs SCFA-producing bacteria (e.g., Propionibacterium), alongside reduced levels of potentially harmful bacteria (Forslund et al., 2015). This shift toward a healthier microbial community structure is closely associated with its metabolic improvement effects. Wu H (Wu et al., 2017) transplanted gut microbiota from T2D patients treated with metformin into germ-free mice. Results showed that mice receiving microbiota altered by metformin exhibited improved glucose tolerance, supporting the notion that gut microbiota changes mediate part of metformin’s antidiabetic effects. Metformin enhances expression of tight junction proteins in the gut, reducing intestinal permeability and thereby decreasing endotoxin (LPS) translocation (Shin et al., 2014). Independently of its hypoglycemic effects, it also promotes secretion of GLP-1 by intestinal L cells, which is crucial for improving postprandial blood glucose control and appetite regulation (Lee and Ko, 2014). This implies that co-administering prebiotics/probiotics capable of further increasing SCFAs production or supplementing specific beneficial bacteria (e.g., Bifidobacterium) alongside metformin may synergistically reinforce the intestinal barrier and amplify GLP-1 effects, thereby more effectively improving IR and weight management (McCreight et al., 2016; Ejtahed et al., 2019).

## 5 Conclusion

Numerous studies have shown that women with PCOS frequently experience gut dysbiosis, primarily characterized by reduced microbial diversity, an imbalance in dominant microbial communities, and disrupted metabolic activity. IR is central to the pathophysiology of PCOS and is closely intertwined with metabolic and reproductive dysfunction. This review comprehensively summarizes the mechanistic pathways through which gut microbiota may influence PCOS-IR, as delineated in preclinical models and supported by observational human studies. However, a critical caveat is warranted: the majority of data establishing causal relationships, such as specific microbial metabolites driving insulin resistance or ovarian dysfunction, derive from animal models and *in vitro* experiments. In humans, the evidence remains predominantly correlational.

Among targeted interventions for the gut microbiome, dietary and lifestyle modifications remain the foundational approaches for improving microbial composition and metabolic status. Probiotics, prebiotics, FMT, and pathogen-specific interventions are transitioning from the laboratory to clinical practice. Of particular note is that the partial efficacy of traditional drugs such as metformin may be achieved precisely through the remodeling of the microbiota-gut-metabolism axis. This suggests that future treatment strategies should shift from single-target approaches to integrated strategies that synergistically regulate the microbiota-host relationship.

Furthermore, the observed association between gut microbiota characteristics and insulin resistance is influenced by various confounding factors, including dietary patterns, obesity, and medications such as metformin and oral contraceptives. Therefore, although microbiota-targeted interventions show promise as adjunctive treatment strategies, their translation into clinical practice requires rigorous human studies that are phenotypically stratified and strictly controlled for confounding factors; Current evidence does not support the use of microbiome-based therapies as an independent treatment for insulin resistance in polycystic ovary syndrome.

However, current understanding of gut microbiota dysbiosis in the pathogenesis of PCOS remains relatively limited, and many research findings require further validation. Most studies employ cross-sectional observational designs, making it difficult to establish causal relationships. Furthermore, clinical trials of interventions such as probiotics and FMT share common issues, including small sample sizes, short durations, significant heterogeneity in strains and dosages, and inadequate methodological quality control, resulting in limited levels of evidence. Furthermore, the composition of the gut microbiota is influenced by multiple factors, including ethnicity, diet, geographic environment, and hormonal status, and there is a lack of standardized microbiota assessment and intervention systems applicable to different PCOS subtypes.

Despite the promising mechanistic insights and preliminary clinical data, it is crucial to acknowledge that the current evidence base does not yet support the routine clinical use of probiotics, prebiotics, or FMT as standard treatments for PCOS-IR. As such, these interventions are best characterized as experimental or adjunctive to foundational lifestyle modifications, and their adoption should be confined to well-designed clinical research settings until more definitive evidence emerges.

In the future, efforts should be made to enhance the translation of basic research findings into clinical applications. As a novel FXR agonist derived from the microbiota, agmatine is synthesized primarily by the enzyme arginine decarboxylase (ADC). In animal studies, ADC inhibitors (such as difluoromethylornithine, DFMO) have been shown to alleviate PCOS-like phenotypes (Yun et al., 2024).Future studies are needed to investigate the safety, pharmacokinetics, and dose-escalation of ADC inhibitors in patients with PCOS-IR.

Microbial metabolites in PCOS-IR exhibit “bidirectional regulation” of FXR, suggesting that an ideal therapeutic strategy should involve localized FXR modulation in the intestinal mucosa. Developing non-absorbable, gut-restricted FXR agonists to avoid hepatic side effects, designing liver-selective FXR antagonists or proteolysis-targeting chimera degraders, and exploring strategies targeting key downstream effector molecules of FXR are essential for achieving precise intervention in signaling pathways. Concurrently, optimizing gut microbiota-based diagnostic and therapeutic strategies for PCOS and developing safe and effective microbial formulations will enhance patient compliance and treatment outcomes.

Current PCOS diagnostics lack objective biomarkers that reflect microbiota-driven IR. Future research should focus on integrated multi-omics analyses and rigorously designed trials to clarify the true efficacy and safety of various microbiota interventions (including single-strain probiotics, probiotic blends, prebiotics, FMT, and small-molecule inhibitors) in improving IR and reproductive and metabolic outcomes. Construct a functional map of the gut microbiota in PCOS-IR patients to identify key bacterial species and metabolites with causal effects. Develop classification models capable of distinguishing different metabolic subtypes and formulate personalized microbiota modulation strategies.

In summary, the gut microbiota has emerged as a crucial window into the pathophysiological mechanisms of PCOS-IR and serves as a strategic cornerstone for its precision treatment. The core path for future development in this field lies in transitioning from mechanistic understanding to clinical validation, and from empirical interventions to evidence-based, personalized microbiome therapy. Only within a framework of interdisciplinary collaboration and technological integration can research findings on the gut microbiota be effectively translated into clinical benefits for PCOS patients.
